# Establishment of IGF-1 and IGFBP-3 continuous reference percentiles from data of healthy children using three kinds of immunoassay systems

**DOI:** 10.1016/j.heliyon.2024.e38245

**Published:** 2024-09-21

**Authors:** Yongseok Jo, Kyungchul Song, Seok-Jae Heo, Junghwan Suh, Hyun Wook Chae, John Hoon Rim, Yongjung Park, Jong Baek Lim, Ho-Seong Kim, Jeong-Ho Kim

**Affiliations:** a Department of Laboratory Medicine, Yongin Severance Hospital, Yonsei University College of Medicine, Yongin, South Korea; b Department of Laboratory Medicine, Severance Hospital, Yonsei University College of Medicine, Seoul, South Korea; c Department of Pediatrics, Yonsei University College of Medicine, Seoul, South Korea; d Department of Biomedical Systems Informatics, Yonsei University College of Medicine, Seoul, South Korea

**Keywords:** Continuous reference percentiles, Reference interval, IGF-1, IGFBP-3, Pediatric, Immunoassay

## Abstract

**Background and aims:**

Appropriate continuous reference intervals (RIs) for serum insulin-like growth factor 1 (IGF-1) and insulin-like growth factor-binding protein 3 (IGFBP-3) are important for diagnosing growth hormone deficiency or excess.

**Material and methods:**

We retrospectively reviewed serum IGF-1 and IGFBP-3 levels in Korean children aged 0–17 years who were diagnosed as healthy during a short stature workup in the outpatient clinics of three hospitals. IGF-1 and IGFBP-3 levels were measured using various immunoassays, including Liaison XL for IGF-1, an immunoradiometric assay (IRMA) for IGFBP-3 (n = 5522), and Immulite 2000 (n = 3036) and cobas e801 (n = 314). We established RIs from the 2.5th to 97.5th percentile RI curves using the lambda–mu–sigma (LMS) method for each sex group.

**Results:**

Pediatric serum continuous IGF-1 and IGFBP-3 reference percentiles by LMS method were found to be immunoassay method-dependent, but aligned relatively well with the manufacturers’ RIs. IGFBP-3 levels displayed notable discrepancies among the different immunoassay methods.

**Conclusion:**

Age- and sex-specific pediatric LMS based continuous reference intervals are method dependent and they should be calculated for dynamic parameters that show variations throughout childhood.

## Highlights

• Continuous reference percentiles were established for serum IGF-1 and IGFBP-3.
• This was conducted using data from healthy children evaluated for short stature.
• Pediatric LMS-based continuous reference intervals were method-dependent.

## 1 Introduction

Insulin-like growth factor 1 (IGF-1) is a hormone produced by the liver that plays an important role in childhood growth. It is mainly stimulated by growth hormone (GH) and has growth promoting effects in various body cells, including skeletal muscles and bone cells [1,2]. The major functions of IGF-binding proteins (IGFBPs) are to transport IGFs, prolong their half-lives and regulate the availability of free IGFs for interactions with IGF receptors, thereby modulating the effects on growth and differentiation [3]. Among the six IGFBPs in humans, IGFBP-3 is the most abundant form in the circulation, and it is also stimulated by GH [4,5].

GH deficiency is the most common pathologic cause of short stature in children, while GH excess can cause gigantism or acromegaly [6,7]. For the diagnosis of GH deficiency or excess, various burdensome tests, including GH provocation test and an oral glucose tolerance test are required. This is because serum GH concentrations vary widely depending on the circadian rhythm, diet, and physical activity levels, and GH deficiency and idiopathic short stature are differentiated using various expensive and/or burdensome diagnostic modalities [8,9]. Serum IGF-1 and IGFBP-3 levels can be used as biomarkers to estimate the production and secretion of GH as they are dependent on GH and remain stable throughout the day [5,10,11]. IGF-1 and IGFBP-3 tests are considered as representative screening tests for children with short height and used in diagnosing growth disorders and monitoring GH replacement therapy results [[12], [13], [14]].

It is important to accurately measure IGF-1 and IGFBP-3 levels, and appropriate reference intervals (RIs) are needed for correct diagnosis [15]. However, various methods and immunoassays to measure these biomarkers are available and often produce inconsistent test results [16,17]. Attempts have been made to standardize serum IGF-1 measurement, and a WHO international standard product (IS 02/254) is available [18,19].

Serum IGF-1 and IGFBP-3 concentrations are influenced by various factors, including sex, age, and ethnicity. Most RIs provided by the manufacturers are based on Caucasian populations, highlighting the need for specific RIs tailored to diverse ethnic groups, such as the Asian population. Korean pediatricians currently largely rely on the RIs derived from a multi-center study involving 1378 healthy Korean children and adolescents using an immunoradiometric assay (IRMA) [3]. However, with the increasing adoption of non-radioisotope assays in clinical laboratories, it becomes crucial to establish and/or validate appropriate RIs for Korean populations using various commercially available immunoassay systems, while considering factors such as sex and age. A recent study successfully established IGF-1 RIs for the Korean adult population using the Liaison chemiluminescence immunoassay [20]; however, the study did not include children and adolescents, nor did it examine IGFBP-3 levels. To address this gap, we aimed to establish pediatric RIs for IGF-1 and IGFBP-3 in healthy pediatric individuals based on data obtained using three different immunoassay systems. This comprehensive approach will provide valuable insights into the variability of IGF-1 and IGFBP-3 levels in the pediatric population, aiding clinicians or laboratorians in providing more accurate and context-specific diagnostic information.

## 2 Material and methods

### 2.1 Data collection

In this retrospective study, medical record data were collected from the ‘Severance Clinical Research Analysis Portal (SCRAP)’ service of Yonsei University Health System. Children and adolescents aged 0–17 years who visited the pediatric endocrinology clinics of three hospitals (Severance Hospital, Gangnam Severance Hospital, and Yongin Severance Hospital) for medical examination and were of normal height (between the 3rd and 97th percentiles) between January 2015 to May 2023 were enrolled, and IGF-1 and IGFBP-3 test results from their first visit were reviewed.

Children and adolescents with short stature (height below the 3rd percentile for age, sex, and ethnicity) and tall stature (height above the 97th percentile for age, sex, and ethnicity) based on the 2017 Korean National Growth Charts [21] were excluded. We also excluded children who had hypertension, diabetes, GH deficiency, or any other endocrine disease history, and children who received human recombinant GH treatment before their first visit. The final numbers of eligible subjects in the three hospitals are listed in Table 1.

**Table 1:** IGF-1 and IGFBP-3 immunoassays manufacturers, numbers of subjects with normal height included in this study, and evaluation periods in the three hospitals.

Hospital	No. of subjects	IGF-1		IGFBP-3		Evalutation Peoriod
		Manufacturer	Traceability	Manufacturer	Traceability	
Severance	8664	Liaison XL (Diasorin)	WHO NIBSC IS 02/254	IRMA (IDS)	NIBSC IS 93/560	January 1, 2015 to May 31, 2023
Gangnam Severance	5407	Immulite 2000 (Siemens)	WHO NIBSC IS 02/254	Immulite 2000 (Siemens)	NIBSC IS 93/560	March 1, 2017 to May 31, 2023
Yongin Severance	410	cobas e801 (Roche)	WHO IS 02/254	cobas e801 (Roche)	IDS iSYS (recombinant glycosylated human IGFBP-3 [1])	September 1, 2021 to May 31, 2023

Abbreviations: WHO, World Health Organization; NIBSC, the National Institute for Biological Standards and Control; IS, Internal Standard, 02/254; IRMA, immunoradiometric assay.

We used the following immunoassay systems for IGF-1 or IGFBP-3 measurements, as summarized in Table 1: (1) the Liaison XL chemiluminescence immunoassay system (DiaSorin S.p.A., Saluggia, Italy), (2) the Immulite 2000 chemiluminescence immunometric assay (Siemens Healthcare Diagnostics, Tarrytown NY, USA), (3) The cobas e801 electrochemiluminescence immunoassay (Roche Diagnostics, Penzberg, Germany), and (4) The IRMA (Immunodiagnostic Systems Ltd., Boldon, UK). The IGF-1 and IGFBP-3 immunoassay methods are also listed in Table 1. We limited the evaluation period to the timeframe during which the new immunoassay systems utilized the same calibrators, such as IS 02/254, to ensure consistency and minimize discordant values arising from variations in traceability, as indicated in Table 1 [22].

The total coefficient of variation of IGF-1 were 5.6–9.6 % for Liaison XL (DiaSorin), 3.0–7.6 % for Immulite 2000 (Siemens), are 1.2–5.8 % for cobas e801 (Roche). The limit of quantification of IGF-1 were 10 ng/mL for Liaison XL (DiaSorin), 24.9 ng/mL for Immulite 2000 (Siemens), 15 ng/mL for cobas e801 (Roche). The reportable range of IGF-1 were 3–1500 ng/mL for Liaison XL (DiaSorin), 15–1000 ng/mL for Immulite 2000 (Siemens), 7–1600 ng/mL for cobas e801 (Roche).

The total coefficient of variation of IGFBP-3 were 6.3–12.4 % for IRMA (IDS), 5.2–7.3 % for Immulite 2000 (Siemens), are 4.49–5.35 % (exceeding the claimed value of 2.1–3.8 %) for cobas e801 (Roche). The limit of quantification of IGFBP-3 were 50 ng/mL for IRMA (IDS), 100 ng/mL for Immulite 2000 (Siemens), 70 ng/mL for cobas e801 (Roche). The reportable range of IGFBP-3 were 50–7550 ng/mL for IRMA (IDS), 100–16,000 ng/mL for Immulite 2000 (Siemens), 70–18,000 ng/mL for cobas e801 (Roche).

### 2.2 Statistical methods

The lambda–mu–sigma (LMS) method summarizes the age-specific distribution of IGF-1 and IGFBP-3 by incorporating skewness, median, and coefficient of variation. Lambda (L), denotes skewness through Box-Cox transform power, mu (M), represents the median, and sigma (S), indicates the coefficient of variation. To establish reference intervals, a natural smoothed spline function is employed to derive smoothed values of lambda, mu, and sigma for each age group. Subsequently, percentiles spanning from the 2.5th to the 97.5th percentiles are computed using these smoothed values for a specific age, ensuring a comprehensive understanding of the distribution across diverse age, sex, and immunoassay groups.

## 3 Results

**Fig. 1:**
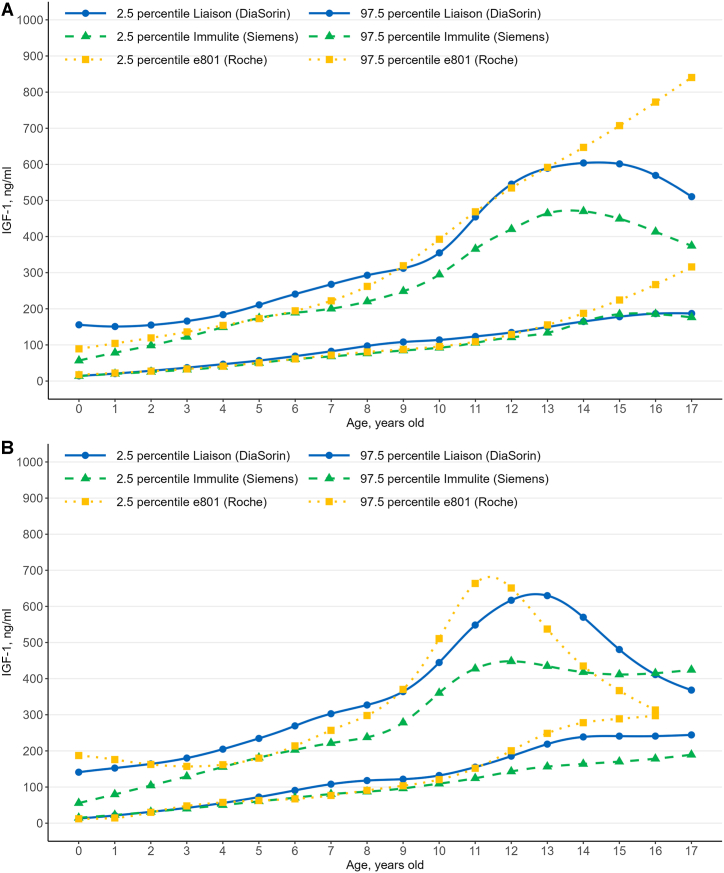
IGF-1 continuous reference intervals for (A) boys and (B) girls computed using the lambda–mu–sigma method, compared in three immunoassay systems.

**Fig. 2:**
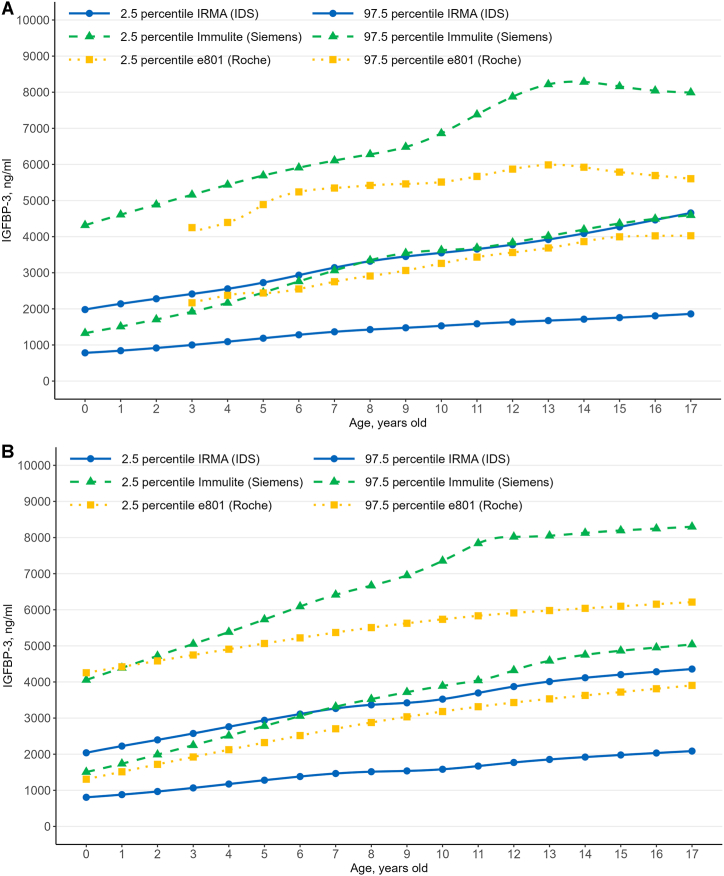
IGFBP-3 continuous reference intervals computed using the lambda–mu–sigma method, (A) boys, (B) girls, based on the three immunoassay systems.

**Fig. 3:**
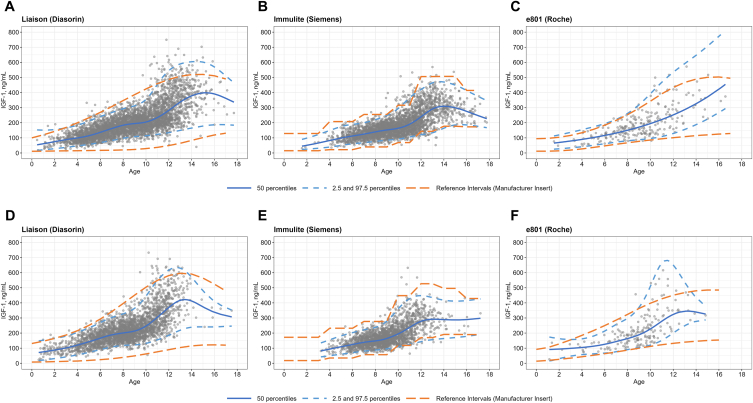
Comparison of IGF-1 reference intervals (RIs) established by the lambda–mu–sigma (LMS) method in boys and girls from three immunoassay systems: (A) Liaison (Diasorin) for boys in Severance Hospital, (B) Immulite (Siemens) for boys in Gangnam Severance Hospital, (C) cobas e801 (Roche) for boys in Yongin Severance Hospital (D) Liaison (Diasorin) for girls in Severance Hospital, (E) Immulite (Siemens) for girls in Gangnam Severance Hospital, (F) cobas e801 (Roche) for girls in Yongin Severance Hospital in contrast to the RIs provided by manufacturers, respectively. Individual data are represented by gray dots, established RIs are indicated by blue lines, and the manufacturer-provided RIs are displayed as orange lines. LMS stands for Lambda for skewness, Mu for median, Sigma for the coefficient of variation.

RIs for IGF-1 and IGFBP-3 continuous reference percentile levels and their corresponding curves established using the LMS method are shown in Table 2, Table 3, Table 4, Table 5, Table 6, Table 7 and Fig. 1, Fig. 2, respectively. Serum IGF-1 levels displayed an age-related increase and peaked at 11–13 years in girls and at 13–14 years in boys, as shown in Fig. 1 and in accordance with the RIs provided by the manufacturers, as illustrated in Fig. 3 and corroborated by another study [23]. Subsequently, these levels began to decline, with slight variation depending on the manufacturer, as shown in Fig. 1. While IGF-1 RIs were generally similar among the three immunoassay systems, there were notable differences in the upper limits of the RIs among the systems, as shown in Fig. 1. In contrast, IGFBP-3 RIs showed great differences among the immunoassay systems.

**Table 2:** IGF-1 continuous reference values (ng/mL) using LMS^a^ data obtained using the Liaison XL (Diasorin) immunoassay system at Severance Hospital.

Boys	No.	Percentiles							Girls	No.	Percentiles						
		2.5	10.0	25.0	50.0	75.0	90.0	97.5			2.5	10.0	25.0	50.0	75.0	90.0	97.5
Age, years									Age, years								
0	2	14.448	22.112	32.185	48.551	72.839	104.511	155.757	0	7	13.012	28.650	45.162	64.715	86.666	110.247	141.051
1	26	20.696	30.878	43.086	60.875	84.342	111.718	150.979	1	33	21.224	37.908	55.054	75.795	98.906	122.649	152.494
2	43	28.492	41.512	55.833	74.850	97.744	122.425	155.080	2	40	31.304	48.263	65.766	87.614	111.823	135.508	164.004
3	143	37.119	52.307	68.151	88.128	111.147	135.191	166.047	3	65	42.290	59.617	77.913	101.690	127.939	152.301	180.196
4	292	46.674	62.404	78.797	99.527	123.751	149.658	183.782	4	149	55.844	74.087	93.859	120.513	149.916	176.073	204.799
5	390	56.793	73.808	91.614	114.271	141.195	170.770	210.902	5	243	72.295	91.821	113.223	142.549	175.023	203.584	234.580
6	437	68.964	89.727	110.772	136.612	166.396	198.393	240.753	6	302	90.733	112.082	135.208	166.546	201.640	233.531	269.309
7	456	82.377	108.168	132.959	161.773	193.417	226.156	267.780	7	363	107.924	131.186	155.751	188.052	224.797	260.356	302.891
8	481	97.223	126.128	153.140	183.684	216.600	250.350	292.845	8	466	117.940	143.014	168.619	200.779	237.879	276.734	327.108
9	535	108.074	136.555	163.660	194.847	229.199	265.326	312.036	9	351	121.718	150.515	179.155	213.518	253.746	299.527	364.091
10	429	113.798	141.834	170.191	204.966	245.823	291.516	354.812	10	306	131.973	167.806	203.672	246.615	297.572	357.438	444.630
11	460	123.453	155.391	189.649	234.565	290.719	356.863	454.335	11	233	155.108	199.512	245.855	304.887	374.380	449.128	548.413
12	284	134.535	176.991	222.561	282.174	354.512	434.861	545.190	12	103	185.488	236.400	292.416	369.447	455.565	532.398	616.909
13	151	149.508	207.261	265.441	336.757	415.625	493.825	589.277	13	26	218.741	270.237	330.165	416.455	505.508	570.888	629.767
14	63	164.817	236.247	302.403	378.020	455.090	525.082	603.908	14	3	238.659	281.211	334.596	412.637	487.236	534.456	569.909
15	20	178.059	255.033	322.856	397.650	470.571	533.526	601.400	15	3	240.920	271.167	312.932	373.685	428.551	460.281	480.596
16	5	186.578	259.101	321.963	390.735	456.468	511.518	569.353	16	1	240.996	261.499	292.788	337.828	377.175	398.955	411.216
17	3	187.033	249.065	302.801	361.813	417.639	463.322	510.362	17	1	244.303	258.447	281.926	315.537	344.483	360.243	368.345
Sum	4220								Sum	2695							

a LMS, lambda–mu–sigma.

**Table 3:** IGF-1 continuous reference values (ng/mL) using LMS^a^ data obtained using the Immulite 2000 (Siemens) immunoassay system at Gangnam Severance Hospital.

Boys	No.	Percentiles							Girls	No.	Percentiles						
		2.5	10.0	25.0	50.0	75.0	90.0	97.5			2.5	10.0	25.0	50.0	75.0	90.0	97.5
Age, years									Age, years								
0	0	–			–			–	0	0	–			–			–
1	2	19.886	23.986	29.185	37.805	49.614	62.142	78.413	1	0	–			–			–
2	1	25.305	31.528	39.056	50.688	65.493	80.321	98.480	2	0	–			–			–
3	5	31.325	40.361	50.782	65.873	83.990	101.482	122.195	3	9	40.249	52.229	64.185	78.818	94.856	110.479	129.258
4	34	39.201	51.015	64.208	82.571	104.046	124.665	149.007	4	20	50.063	63.396	76.977	93.974	113.062	132.098	155.528
5	117	50.070	63.596	78.467	98.838	122.718	146.152	174.485	5	91	60.401	75.131	90.295	109.520	131.440	153.641	181.416
6	225	60.623	75.640	91.525	112.349	136.300	160.077	189.226	6	204	70.541	86.765	103.402	124.430	148.350	172.539	202.768
7	324	68.252	86.253	103.916	125.118	148.407	171.668	200.319	7	337	80.480	98.195	116.207	138.792	164.272	189.848	221.579
8	452	76.499	97.858	117.613	139.644	163.445	188.331	220.244	8	433	87.348	105.705	124.516	148.320	175.466	203.017	237.605
9	424	84.525	107.259	128.518	152.217	178.653	208.206	248.724	9	365	96.220	115.863	136.723	164.210	197.067	232.072	278.390
10	444	92.188	113.685	135.889	163.755	197.602	237.112	294.703	10	261	109.349	133.029	159.088	194.850	239.703	289.972	360.371
11	417	105.821	128.218	153.779	190.639	238.186	291.335	365.609	11	162	124.338	156.637	191.323	237.307	292.354	350.818	427.620
12	256	120.943	152.686	188.564	239.451	299.063	355.232	420.280	12	76	143.113	185.684	227.573	278.161	332.866	385.517	448.079
13	146	133.714	183.495	232.127	291.028	351.918	405.482	464.050	13	27	156.486	201.942	243.758	291.303	339.850	384.311	434.774
14	88	164.031	213.590	258.660	308.861	360.829	410.768	469.932	14	9	163.887	206.860	245.747	289.358	333.341	373.208	418.043
15	31	185.105	223.500	259.143	299.170	342.848	389.079	449.240	15	4	170.290	209.760	246.012	287.202	329.256	367.776	411.515
16	21	185.605	214.389	242.335	275.372	313.282	355.319	413.259	16	2	178.411	215.074	249.531	289.527	331.221	370.122	415.064
17	5	176.337	197.794	219.701	247.382	280.815	319.034	374.348	17	1	189.382	223.980	257.130	296.351	338.051	377.674	424.272
Sum	2992								Sum	2001							

a LMS, lambda–mu–sigma.

**Table 4:** IGF-1 continuous reference values (ng/mL) using LMS^a^ data obtained using the cobas e801 (Roche) immunoassay system at Yongin Severance Hospital.

Boys	No.	Percentiles							Girls	No.	Percentiles						
		2.5	10.0	25.0	50.0	75.0	90.0	97.5			2.5	10.0	25.0	50.0	75.0	90.0	97.5
Age, years									Age, years								
0	0	–			–			–	0	0	–			–			–
1	2	22.208	34.781	46.773	60.737	75.262	88.751	104.239	1	2	14.495	36.972	62.048	91.342	120.751	147.009	176.049
2	0	27.451	40.822	53.854	69.350	85.793	101.326	119.436	2	2	29.987	51.229	71.207	94.012	117.280	138.538	162.595
3	4	33.594	47.764	61.842	78.903	97.356	115.081	136.074	3	2	47.691	64.381	80.238	98.755	118.120	136.211	157.111
4	12	41.204	56.158	71.226	89.776	110.176	130.080	154.017	4	6	57.487	72.519	87.099	104.494	123.101	140.849	161.769
5	11	50.633	66.327	82.279	102.142	124.286	146.186	172.892	5	5	62.685	78.288	93.874	113.024	134.137	154.844	179.924
6	21	61.270	77.863	94.848	116.212	140.323	164.476	194.332	6	13	67.321	84.755	102.860	125.996	152.579	179.682	213.802
7	33	71.772	89.789	108.442	132.219	159.477	187.222	222.096	7	21	76.776	96.043	116.685	144.003	176.669	211.345	256.898
8	34	80.589	100.920	122.412	150.430	183.358	217.703	261.980	8	32	90.744	111.254	133.600	163.859	201.139	242.064	297.970
9	39	87.662	111.125	136.698	171.147	213.123	258.497	319.213	9	34	103.525	126.561	152.441	188.851	235.944	290.551	370.145
10	29	95.692	122.501	152.650	194.716	248.097	308.256	392.488	10	25	120.539	147.977	180.065	227.614	293.517	376.498	510.887
11	21	108.824	138.615	172.748	221.530	285.372	359.857	468.491	11	14	152.061	183.873	221.567	278.719	361.021	470.507	663.453
12	14	128.971	161.313	198.482	252.034	323.131	407.731	534.488	12	6	200.455	232.779	269.724	323.602	397.858	492.335	650.917
13	7	155.478	190.151	229.771	286.736	362.555	453.472	591.665	13	5	248.630	275.744	304.918	344.563	394.611	452.195	537.249
14	6	187.455	224.443	266.343	326.213	405.652	501.023	646.960	14	1	278.117	296.750	315.629	339.574	367.369	396.499	434.922
15	2	224.340	263.816	308.166	371.124	454.299	554.072	707.329	15	0	288.837	299.749	310.284	322.954	336.773	350.332	366.928
16	2	267.032	309.139	356.055	422.176	509.019	612.858	772.419	16	0	297.474	300.057	302.412	305.079	307.800	310.296	313.142
17	0	315.913	360.667	410.105	479.219	569.305	676.402	840.390	17	0	NA			NA			NA
Sum	237								Sum	168							

a LMS, lambda–mu–sigma.

**Table 5:** IGFBP-3 continuous reference values (ng/mL) using LMS^a^ data obtained using the immunoradiometric assay (Immunodiagnostic Systems) system at Severance Hospital.

Boys	No.	Percentiles							Girls	No.	Percentiles						
		2.5	10.0	25.0	50.0	75.0	90.0	97.5			2.5	10.0	25.0	50.0	75.0	90.0	97.5
Age, years									Age, years								
0	2	782.476	1025.648	1175.411	1264.875	1360.079	1550.126	1980.750	0	7	806.069	1030.709	1176.153	1266.853	1365.382	1564.573	2038.708
1	26	842.168	1068.180	1232.709	1358.467	1495.530	1717.058	2139.936	1	31	880.073	1097.896	1259.084	1382.742	1520.020	1751.929	2224.205
2	43	916.538	1122.461	1294.369	1456.268	1636.822	1880.471	2280.100	2	40	966.360	1175.224	1347.520	1504.744	1682.571	1939.883	2396.281
3	141	1000.134	1186.830	1361.962	1558.389	1782.133	2041.549	2413.500	3	64	1065.503	1266.554	1447.988	1638.836	1857.966	2136.311	2574.396
4	292	1090.993	1262.324	1439.705	1668.170	1933.306	2206.203	2555.280	4	148	1172.897	1368.252	1558.119	1781.645	2041.251	2337.972	2760.347
5	390	1186.700	1352.768	1535.763	1791.128	2091.504	2381.253	2727.456	5	244	1279.055	1469.413	1666.550	1920.553	2218.133	2530.188	2937.825
6	435	1282.214	1455.215	1648.985	1924.906	2251.786	2564.179	2933.469	6	304	1380.973	1569.536	1774.217	2054.792	2385.317	2711.657	3111.792
7	457	1365.287	1554.212	1761.801	2050.603	2393.228	2730.481	3142.400	7	364	1465.193	1657.279	1870.022	2169.052	2521.893	2861.480	3266.399
8	481	1426.880	1635.610	1856.936	2150.979	2498.897	2858.184	3320.313	8	465	1514.556	1713.901	1933.272	2239.231	2599.640	2948.465	3367.052
9	532	1476.299	1697.880	1928.961	2229.145	2583.686	2958.099	3451.132	9	348	1536.800	1745.233	1969.631	2274.169	2631.609	2986.019	3422.658
10	426	1530.039	1752.592	1989.020	2303.937	2676.536	3060.121	3551.899	10	303	1582.248	1801.416	2033.481	2341.802	2702.600	3066.981	3524.770
11	455	1586.953	1811.199	2053.905	2385.180	2777.058	3169.122	3656.581	11	232	1671.084	1897.796	2139.857	2464.994	2845.559	3225.296	3696.367
12	280	1635.450	1873.774	2127.950	2468.358	2868.258	3271.589	3777.273	12	102	1770.744	2000.206	2250.989	2597.922	3004.832	3398.980	3872.594
13	150	1675.528	1943.866	2215.875	2556.340	2950.902	3369.301	3921.034	13	24	1853.948	2084.089	2341.138	2706.205	3135.103	3539.602	4011.528
14	62	1714.098	2026.230	2320.845	2654.607	3034.692	3470.137	4087.398	14	3	1920.217	2150.394	2411.906	2790.735	3236.253	3647.866	4117.022
15	21	1757.424	2123.235	2442.582	2765.774	3127.142	3579.247	4271.060	15	3	1977.908	2207.384	2472.251	2862.764	3322.382	3739.218	4204.204
16	5	1806.442	2234.069	2578.105	2887.163	3226.666	3693.110	4463.766	16	0	2032.384	2260.672	2528.355	2929.758	3402.520	3823.671	4283.558
17	2	1860.876	2358.522	2725.262	3015.406	3328.814	3805.157	4657.083	17	1	2085.465	2312.254	2582.451	2994.336	3479.748	3904.692	4358.921
Sum	4200								Sum	2695							

a LMS, lambda–mu–sigma.

**Table 6:** IGFBP-3 continuous reference values (ng/mL) using LMS^a^ data obtained using the Immulite 2000 (Siemens) immunoassay system at Gangnam Severance Hospital.

Boys	No.	Percentiles							Girls	No.	Percentiles						
		2.5	10.0	25.0	50.0	75.0	90.0	97.5			2.5	10.0	25.0	50.0	75.0	90.0	97.5
Age, years									Age, years								
0	0	–			–			–	0	0	–			–			–
1	1	1510.632	1972.673	2411.959	2928.137	3474.773	3994.523	4609.401	1	0	–			–			–
2	1	1706.435	2194.019	2651.106	3182.263	3739.698	4266.364	4886.735	2	0	–			–			–
3	4	1919.689	2429.860	2901.399	3443.449	4007.592	4537.792	5160.564	3	6	2247.869	2795.400	3255.894	3742.773	4208.870	4613.725	5052.275
4	22	2165.177	2693.413	3174.703	3722.099	4287.381	4816.309	5436.581	4	13	2510.021	3038.104	3496.973	3994.505	4481.165	4911.195	5383.953
5	92	2453.324	2989.274	3470.411	4011.816	4566.816	5084.288	5690.944	5	65	2780.045	3292.613	3750.552	4258.671	4766.169	5222.468	5731.943
6	175	2761.739	3295.211	3767.034	4292.486	4827.627	5325.450	5909.943	6	152	3057.562	3555.597	4011.807	4529.476	5057.725	5541.660	6091.481
7	249	3063.371	3590.215	4049.262	4555.433	5068.117	5544.726	6106.397	7	229	3315.415	3798.589	4250.385	4773.412	5318.214	5826.945	6415.926
8	325	3346.279	3864.344	4308.754	4793.983	5283.210	5738.471	6278.336	8	278	3525.708	3996.901	4442.715	4965.983	5520.189	6047.022	6669.307
9	327	3544.128	4073.462	4520.021	5002.752	5487.691	5940.294	6481.982	9	277	3718.083	4196.326	4645.603	5173.522	5738.525	6286.037	6951.860
10	350	3625.543	4220.430	4712.803	5239.245	5766.411	6260.789	6859.882	10	208	3890.253	4420.710	4896.295	5441.779	6025.997	6607.708	7355.402
11	310	3689.026	4384.607	4947.036	5540.482	6132.673	6691.591	7379.688	11	135	4042.970	4667.505	5188.356	5761.041	6368.758	6990.064	7840.490
12	188	3835.599	4618.539	5233.975	5873.194	6508.746	7113.755	7873.757	12	58	4322.950	4944.288	5472.001	6053.447	6659.264	7255.001	8021.045
13	109	4014.482	4855.221	5494.329	6146.072	6791.929	7413.998	8215.945	13	24	4587.049	5171.667	5689.701	6267.700	6857.308	7406.365	8051.299
14	60	4196.807	5043.306	5661.858	6279.442	6889.907	7487.390	8283.828	14	9	4751.799	5341.375	5863.772	6441.200	7018.299	7539.828	8127.757
15	20	4365.320	5181.816	5750.987	6305.356	6852.473	7399.585	8160.832	15	0	4865.796	5473.868	6003.863	6579.760	7144.289	7644.299	8195.589
16	5	4495.079	5291.780	5816.435	6312.300	6801.196	7303.939	8042.441	16	1	4953.694	5583.933	6121.190	6693.839	7245.001	7725.364	8246.946
17	0	4595.265	5396.487	5888.770	6336.927	6778.567	7249.561	7990.834	17	0	5037.837	5690.851	6234.412	6802.714	7340.388	7802.418	8297.839
Sum	2238								Sum	2695							

a LMS, lambda–mu–sigma.

**Table 7:** IGFBP-3 continuous reference values (ng/mL) using LMS^a^ data obtained using the cobas e801 (Roche) immunoassay system at Yongin Severance Hospital.

Boys	No.	Percentiles							Girls	No.	Percentiles						
		2.5	10.0	25.0	50.0	75.0	90.0	97.5			2.5	10.0	25.0	50.0	75.0	90.0	97.5
Age, years									Age, years								
0	0								0	0	–			–			–
1	2								1	2	1513.135	2016.321	2466.586	2966.862	3467.139	3917.403	4420.589
2	0	1993.104	2763.254	2763.254	2763.254	2763.254	2763.254	3910.795	2	2	1718.617	2214.621	2658.460	3151.596	3644.732	4088.571	4584.575
3	4	2218.250	2330.722	2573.814	3039.775	3594.841	3977.974	4185.480	3	2	1922.441	2411.365	2848.867	3334.964	3821.061	4258.563	4747.487
4	12	2375.469	2503.265	2757.708	3237.170	3795.909	4174.722	4393.909	4	6	2123.523	2605.468	3036.724	3515.882	3995.040	4426.296	4908.241
5	11	2440.749	2769.435	3092.850	3486.695	3922.163	4355.219	4889.200	5	5	2321.590	2796.655	3221.755	3694.073	4166.390	4591.491	5066.555
6	21	2552.053	3072.666	3439.723	3720.380	4017.495	4463.113	5240.380	6	13	2516.410	2984.693	3403.725	3869.300	4334.875	4753.907	5222.190
7	32	2752.367	3199.014	3564.975	3917.128	4290.156	4727.878	5347.855	7	20	2704.407	3166.004	3579.053	4037.981	4496.909	4909.959	5371.556
8	32	2910.910	3329.722	3691.038	4067.161	4461.926	4879.979	5420.972	8	32	2877.930	3332.936	3740.088	4192.463	4644.838	5051.990	5506.996
9	38	3063.546	3443.446	3794.126	4194.075	4609.833	5002.518	5461.655	9	34	3035.465	3483.974	3885.312	4331.227	4777.142	5178.480	5626.989
10	28	3259.851	3573.521	3905.009	4344.118	4796.341	5156.136	5512.544	10	26	3181.661	3623.765	4019.371	4458.919	4898.466	5294.072	5736.176
11	20	3434.414	3753.079	4087.554	4527.077	4974.193	5324.999	5668.234	11	14	3315.280	3751.070	4141.026	4574.296	5007.566	5397.523	5833.313
12	14	3561.909	3949.699	4303.985	4706.541	5111.275	5471.139	5869.104	12	6	3430.974	3860.539	4244.926	4672.008	5099.089	5483.476	5913.042
13	7	3687.233	4103.464	4461.995	4843.549	5223.633	5578.106	5986.347	13	4	3532.941	3956.370	4335.267	4756.248	5177.229	5556.125	5979.554
14	6	3863.437	4156.921	4477.110	4909.802	5336.434	5644.467	5921.158	14	1	3628.035	4045.416	4418.900	4833.868	5248.836	5622.320	6039.702
15	2	3996.433	4165.515	4451.215	4913.729	5364.640	5631.947	5786.231	15	0	3720.626	4132.039	4500.183	4909.218	5318.253	5686.397	6097.810
16	2	4023.188	4164.034	4439.688	4885.128	5315.442	5567.268	5691.867	16	0	3812.936	4218.394	4581.208	4984.321	5387.434	5750.249	6155.706
17	0	4025.835	4160.768	4424.347	4847.204	5252.465	5488.293	5604.333	17	0	3905.246	4304.748	4662.233	5059.424	5456.616	5814.101	6213.603
Sum	231								Sum	167							

a LMS, lambda–mu–sigma.

**Fig. 4:**
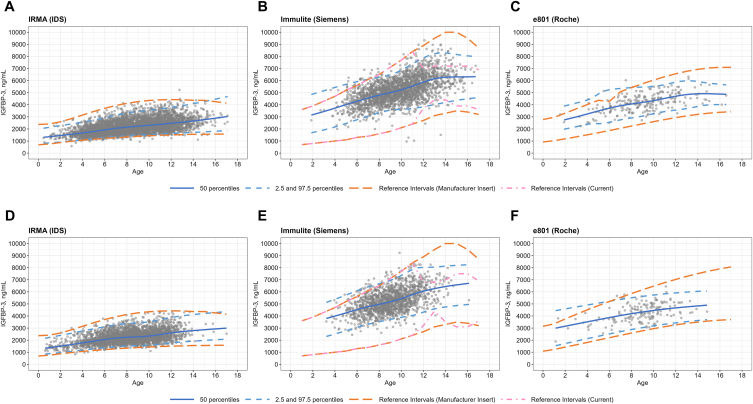
Comparison of IGFBP-3 reference intervals (RIs) established by the lambda–mu–sigma (LMS) method in boys and girls from three immunoassay systems: (A) Immunoradiometric assay (IRMA, IDS) for boys in Severance Hospital, (B) Immulite (Siemens) for boys in Gangnam Severance Hospital, (C) cobas e801 (Roche) for boys in Yongin Severance Hospital (D) Immunoradiometric assay (IRMA, IDS) for girls in Severance Hospital, (E) Immulite (Siemens) for girls in Gangnam Severance Hospital, (F) e801 (Roche) for girls in Yongin Severance Hospital, in contrast to the RIs provided by manufacturers, respectively. Individual data are represented by gray dots, established RIs are indicated by blue lines, and the manufacturer-provided RIs are displayed as orange lines. Pink dashed lines represent the current reference intervals established about 19 years ago at Gangnam Severance Hospital. LMS stands for Lambda for skewness, Mu for median, Sigma for the coefficient of variation.

Next, we compared the IGF-1 RIs with those suggested by manufacturers. For both boys and girls, the lower limits of the IGF-1 RIs based on the Liaison immunoassay were higher than manufacturers' lower limits (Fig. 3). This finding aligns with previously reported results in a Korean adult population [20]. For the other immunoassays, the RIs were generally similar to the manufacturer's limits. However, there was a discrepancy in the limits for the cobas e801 assay in subjects older than 11 years, likely due to the small sample size (Fig. 3). Regarding IGFBP-3, the lower limits determined by the Immulite 2000 immunoassay were higher than the manufacturer's lower limits for both boys and girls. For the other immunoassays, the RIs were similar to manufacturers' limits (Fig. 4).

When comparing the newly derived IGF-1 RIs with previously reported RIs based on the IRMA method [3], the limits were largely similar. However, the reported upper RI limits were higher than those established in the present study and those suggested by the manufacturer, particularly for girls. Similarly, when comparing the newly derived IGFBP-3 RIs with previously reported RIs based on the IRMA method [3], the limits were largely comparable, although the reported limits were slightly higher than the newly derived ones and those suggested by the manufacturer.

When we compared the IGF-1 RIs based on Immulite 2000 with those reported in a Chinese study that used the same immunoassay system [23], the reported upper RIs were slightly higher than those established in the current study, whereas the lower RIs were similar.

## 4 Discussion

This study established continuous reference percentiles for IGF-1 and IGFBP-3 for three kinds of immunoassay systems, using the LMS method, showing that IGF-1 levels increase with age, peaking in early adolescence before declining, with variations depending on the manufacturer. While IGF-1 RIs were generally similar across different immunoassay systems, the lower RIs limit were higher than the manufacturer-provided limits. In contrast, IGFBP-3 RIs varied greatly among the immunoassays. RIs provided by the manufacturers have certain limitations, including: (1) Most RIs are based on central 95 % range without smoothed centile curves, while some use M −2SD (standard deviation) to M +2SD without assuming a normal distribution (see Supplementary Tables 1, 2, 3, and 4). Some RIs use smoothed centile curves but often apply the Royston-Wright method, which differs from our LMS method (see Supplementary Tables 1 and 2); (2) The sample sizes used to establish these RIs is often insufficient. For example, only 448 “normal” subjects were used to establish IGFBP-3 RIs for all individuals aged 0–95 years old using the immunoradiometric assay (IDS) (see Supplementary Tables 1 and 2); and (3) Each manufacture recommends verifying the transferability of the RIs or establishing them based on the specific population served, which is particularly challenging for pediatric population (see Supplementary Tables 1, 2, 3, and 4).

Because of their stability and low diurnal variation compared to GH, IGF-1 and IGFBP-3 levels are used to screen for GH secretory disorders, including gigantism, acromegaly, and GH deficiency, and to monitor GH treatment for short stature [11]. In children treated with human recombinant GH for short stature, risk of serious adverse reaction including tumor increases in patients with IGF-1 level above 2 standard deviation score. However, elevated IGF-1 levels can be counterbalanced by IGFBP-3, thus no definitive tumorigenesis was observed over the 5-year among children with elevated levels of both IGF-1 and IGFBP-3 in a Korean study [24]. Therefore, reduction in GH dose should be considered in children with short stature who treated with human recombinant GH if serum IGF-1 levels increase above the RI and IGFBP-3 levels are low [25]. In addition, an oral glucose tolerance test should be considered in individuals with tall stature who have IGF-1 level above RI to diagnose acromegaly [26]. Furthermore, low IGF-1 level suggests GH deficiency in screening of short stature in chlidren, while high IGF-1 levels is reportedly related cancer or mortality [5,25].

Attempts to standardize serum IGF-1 have been successful when using WHO international standard product (IS 02/254) rather than IS 87/158, as the latter has been recognized to have an incorrectly assigned concentration [16,18]. RIs show moderate to good agreement among IGF-1 immunoassay methods when using IS 02/254 [1,[27], [28]].

Differences in IGF-1 levels according to race/ethnicity have been repeatedly reported [[29], [30], [31], [32]]. Accordingly, IGF-1 reference intervals have been reported in Japanese [33], Koreans [3,20], and Chinese [23,34]. Although direct sampling techniques are ideal for establishing RIs, these are difficult to apply to pediatric populations. Moreover, indirect approaches using pathology samples have potential advantages as they are faster, cheaper, and do not involve patient inconvenience, discomfort, or the risk associated with generating new patient health information. Further, indirect methods use the same preanalytical and analytical techniques for patient management and therefore, they were recommended by a working group of the International Federation of Clinical Chemistry Committee on Reference Intervals and Decision Limits [35]. We used an indirect method to establish pediatric RIs for IGF-1 and IGFBP-3 derived from pediatric patients visiting for a short stature workup. Approximately 89.4 % of the subjects with the chief complaint of a short stature who visited the outpatient clinic were of normal height because Koreans often worry about the height of their children even when it is within normal limits [36]. Moreover, it has been reported that Korean adolescents with relative short stature have higher rates of psychological distress including suicide ideation [37]. Some of the authors of this article had previous experience in successfully establishing RIs using biochemistry tests with a similar approach [38]. In this study, we successfully established pediatric age- and sex-specific continuous reference percentiles for IGF-1 and IGFBP-3 for healthy children with normal height who visited pediatric endocrinology clinics.

The newly established IGF-1 RI limits were generally comparable across the three immunoassay systems. However, having similar IGF-1 RIs across the different immunoassay platforms does not necessarily ensure effective inter-platform monitoring of the IGF-1 assay. This is highlighted by reported instances of less difference of RIs despite significant discordance in IGF-1 levels among four different platforms using the same 02/254 WHO standard as calibrator [39]. Therefore, caution should be exercised when considering a switch from one platform to another for monitoring IGF-1 levels [39]. It is worth noting that the lower limit of the RIs for IGF-1, as measured using the Liaison XL assay, was observed to be higher than the manufacturer's reported values for both boys and girls. This observation is consistent with previous findings in adults [20]. The RIs established in this study tended to align with those from the manufacturers, with only minor differences in the lower limits. These differences are unlikely to result in clinical issues, as the lower limits are used in the screening of GH deficiency, and a diagnosis should be confirmed through GH stimulation tests.

IGFBP-3 levels varied widely among the immunoassay systems, showing a great degree of method dependence. These findings highlight the necessity of standardization and harmonization of IGFBP-3. However, while an international standard is available for IGFBP-3 (NIBSC IS 93/560), it is not suitable for use in the standardization of various immunoassays, as indicated by estimates obtained for the ampoule content of the non-glycosylated material [1,40].

The IGFBP-3 RIs established using the IRMA or cobas e801 were generally comparable to manufacturers' RI limits. However, the IGFBP-3 RI lower limit established using Immulite 2000 in Gangnam Severance Hospital was higher than manufacturer's lower limit. This hospital used IGFBP-3 RI limits for 12–17-year-olds that had been established in-house 19 years ago, and their RI limits were slightly closer to the newly established limits as shown in Fig. 4.

The slightly higher upper limit of the IGF-1 RI established based on the Immulite 2000 assay in a previous Chinese study [23], compared to that established using the same immunoassay system in our study, may suggest ethnic differences or influences from environmental factors, such as nutrition, and highlight the necessity of the validation of RIs in each population.

This study had some limitations. Firstly, the absence of RIs stratified by Tanner stage is a notable limitation due to the unavailability of relevant data. Prospective studies with more comprehensive data could address the gap and offer insights into age-specific variations. Secondly, the limited number of subjects might hinder the establishment of IGF-1 and IGFBP-3 RIs for teenagers using Roche e801 assay and the LMS method. Moreover, IGF-1 and IGFBP-3 data for some age groups (younger than 4 years old or older than 14 years old) are insufficient, which introduces greater uncertainty in the continuous reference percentiles for these age groups. This underscores the importance of larger sample sizes in future studies targeting this age group.

In conclusion, we established IGF-1 and IGFBP-3 continuous reference percentiles for pediatric populations aged 0–17 years using the LMS method based on three immunoassay systems. This will contribute to clinical interpretation for an accurate diagnosis and follow-up of pediatric population.

## Research funding

This research did not receive any specific grant from funding agencies in the public, commercial, or not-for-profit sectors.

## Ethical statement

This study was conducted in accordance with the Declaration of Helsinki and approved by the Institutional Review Board of Yongin Severance Hospital, Yonsei University Health System, Korea (IRB No. 9-2023-0073, date of approval: June 23, 2023).

## Informed consent

Patient consent was waived because of the retrospective nature of the study. Patient data were encrypted to ensure pseudonymity.

## Data availability statement

The data used in this study are not publicly available due to confidentiality and contractual agreements between Yonsei University Health System (YUHS) and the researchers. However, pseudonymized, limited data may be provided by the corresponding authors upon reasonable request, subject to written approval from YUHS.

## CRediT authorship contribution statement

**Yongseok Jo:** Writing – review & editing, Writing – original draft, Visualization, Methodology, Investigation, Formal analysis, Data curation, Conceptualization. **Kyungchul Song:** Writing – review & editing, Supervision, Project administration, Methodology, Investigation, Formal analysis, Data curation, Conceptualization. **Seok-Jae Heo:** Writing – review & editing, Visualization, Methodology, Investigation, Formal analysis, Conceptualization. **Junghwan Suh:** Writing – review & editing, Investigation, Conceptualization. **Hyun Wook Chae:** Writing – review & editing, Methodology, Conceptualization. **John Hoon Rim:** Writing – review & editing, Methodology, Investigation, Conceptualization. **Yongjung Park:** Writing – review & editing, Visualization, Methodology, Investigation, Conceptualization. **Jong Baek Lim:** Writing – review & editing, Methodology, Conceptualization. **Ho-Seong Kim:** Writing – review & editing, Supervision, Project administration, Investigation, Conceptualization. **Jeong-Ho Kim:** Writing – review & editing, Visualization, Supervision, Project administration, Methodology, Investigation, Formal analysis, Data curation, Conceptualization.

## Declaration of competing interest

The authors declare that they have no known competing financial interests or personal relationships that could have appeared to influence the work reported in this paper.
