# Supplementary material for: Establishment of IGF-1 and IGFBP-3 continuous reference percentiles from data of healthy children using three kinds of immunoassay systems
Source: Heliyon. 2024 Sep 21;10(19):e38245. doi: 10.1016/j.heliyon.2024.e38245 (PMC11472241; doi:10.1016/j.heliyon.2024.e38245)
Supplement: Multimedia component 1 [file mmc1.docx]

Supplementary materials

| Supplementary Table 1. IGF-1 (unit, ng/mL) male reference intervals according to manufacturers | | | | | |
| --- | --- | --- | --- | --- | --- |
| Liaison XL (DiaSorin)^1)^ | | Immulite 2000 (Siemens)^2)^ | | e801 (Roche)^3)^ | |
| Age (yr) | RIs | Age (yr) | RIs | Age (yr) | RIs |
| 0 | 11–100 | 0–3 | <15–129 | 0 | 11.8–94.6 |
| 1 | 12–120 |  |  | 1 | 11.8–96.4 |
| 2 | 13–143 |  |  | 2 | 13.9–104 |
| 3 | 14–169 |  |  | 3 | 18.9–116 |
| 4 | 15–200 | 4–6 | 22–208 | 4 | 26.8–134 |
| 5 | 16–233 |  |  | 5 | 36.6–156 |
| 6 | 17–269 |  |  | 6 | 47.1–184 |
| 7 | 18–307 | 7–9 | 40–255 | 7 | 57.5–216 |
| 8 | 20–347 |  |  | 8 | 67.5–254 |
| 9 | 23–386 |  |  | 9 | 76.9–296 |
| 10 | 29–424 | 10–11 | 69–316 | 10 | 85.7–343 |
| 11 | 37–459 |  |  | 11 | 93.9–392 |
| 12 | 49–487 | 12–13 | 143–506 | 12 | 101–434 |
| 13 | 64–508 |  |  | 13 | 108–467 |
| 14 | 83–519 | 14–15 | 177–507 | 14 | 115–489 |
| 15 | 102–520 |  |  | 15 | 120–501 |
| 16 | 119–511 | 16–18 | 173–414 | 16 | 125–503 |
| 17 | 131–490 |  |  | 17 | 129–495 |
| 18 | 137–461 |  |  | 18 | 132–476 |
| 19 | 137–428 | 19–21 | 117–323 | 19 | 134–450 |
| 20 | 133–395 |  |  | 20 | 136–421 |
| 21 | 127–364 |  |  | 21 | 137–394 |
| ^1)^ LIAISON IGF-I pediatric reference ranges, stratified by age and gender, were established based on data from 2,813 pediatric subjects from an apparently healthy population. The central 95% range values were determined following CLSI guideline C28-A3, using the centile smoothing Royston-Wright method. It is recommended that each laboratory establishes its own ranges tailored to the specific population it serves. This information is detailed in the LIAISON IFG-1 insert (REF 313231, dated 2015-10-13, DiaSorin).  ^2)^ A reference range study was conducted using the IMMULITE 2000 IGF-I kit on 1804 pediatric and adult samples from an apparently healthy population. This study is documented in Immulite 2000 IGF-1 insert (PIL2KIGF-4, dated 2018-07-02, Siemens)  ^3)^ Expected values, ranging from the 2.5 percentile to the 97.5 percentile, were obtained in a clinical study (CIM RD002173) involving over 1,400 subjects aged 17 years or younger. Each laboratory should assess the transferability of these expected values to its own patient population and, if necessary, establish its own reference ranges. This is outlined in the Elecsys IGF-1 insert (document 07475918190, dated April, 2018, Roche Diagnostics). | | | | | |

| Supplementary Table 2. IGF-1 (unit, ng/mL) female reference intervals according to manufacturers | | | | | |
| --- | --- | --- | --- | --- | --- |
| Liaison XL (DiaSorin) ^1)^ | | Immulite 2000 (Siemens)^2)^ | | e801 (Roche)^3)^ | |
| Age (yr) | RIs | Age (yr) | RIs | Age (yr) | RIs |
| 0 | 8–131 | 0–3 | 18–172 | 0 | 13.8–92.0 |
| 1 | 9–146 |  |  | 1 | 18.7–104 |
| 2 | 11–165 |  |  | 2 | 26.1–128 |
| 3 | 13–187 |  |  | 3 | 34.2–155 |
| 4 | 15–216 | 4–6 | 35–232 | 4 | 43.2–185 |
| 5 | 19–251 |  |  | 5 | 53.0–216 |
| 6 | 24–293 |  |  | 6 | 63.6–250 |
| 7 | 30–342 | 7–9 | 57–277 | 7 | 75.0–286 |
| 8 | 39–396 |  |  | 8 | 87.3–324 |
| 9 | 49–451 |  |  | 9 | 99.9–363 |
| 10 | 62–504 | 10–11 | 118–448 | 10 | 112–398 |
| 11 | 76–549 |  |  | 11 | 123–427 |
| 12 | 90–581 | 12–13 | 170–527 | 12 | 132–451 |
| 13 | 104–596 |  |  | 13 | 140–468 |
| 14 | 115–591 | 14–15 | 191–496 | 14 | 146–480 |
| 15 | 121–564 |  |  | 15 | 151–485 |
| 16 | 122–524 | 16–18 | 190–429 | 16 | 154–485 |
| 17 | 120–479 |  |  | 17 | 156–479 |
| 18 | 117–436 |  |  | 18 | 156–466 |
| 19 | 113–399 | 19–21 | 117–323 | 19 | 155–449 |
| 20 | 109–372 |  |  | 20 | 152–429 |
| 21 | 107–351 |  |  | 21 | 148–410 |
| ^1)^ LIAISON IGF-I pediatric reference ranges, stratified by age and gender, were established based on data from 2,813 pediatric subjects from an apparently healthy population. The central 95% range values were determined following CLSI guideline C28-A3, using the centile smoothing Royston-Wright method. It is recommended that each laboratory establishes its own ranges tailored to the specific population it serves. This information is detailed in the LIAISON IFG-1 insert (REF 313231, dated 2015-10-13, DiaSorin).  ^2)^ A reference range study was conducted using the IMMULITE 2000 IGF-I kit on 1804 pediatric and adult samples from an apparently healthy population. This study is documented in Immulite 2000 IGF-1 insert (PIL2KIGF-4, dated 2018-07-02, Siemens)  ^3)^ Expected values, ranging from the 2.5 percentile to the 97.5 percentile, were obtained in a clinical study (CIM RD002173) involving over 1,400 subjects aged 17 years or younger. Each laboratory should assess the transferability of these expected values to its own patient population and, if necessary, establish its own reference ranges. This is outlined in the Elecsys IGF-1 insert (document 07475918190, dated April, 2018, Roche Diagnostics). | | | | | |

| Supplementary Table 3. IGFBP-3 (unit, ng/mL) male reference intervals according to manufacturers | | | | | |
| --- | --- | --- | --- | --- | --- |
| IRMA (IDS)^1)^ | | Immulite 2000 (Siemens)^2)^ | | e801 (Roche)^3)^ | |
| Age (yr) | RIs | Age (yr) | RIs | Age (yr) | RIs |
| 0 | 689–2370 | - | - | 0 | 919–2782 |
| 1 | 756–2412 | 1 | 700–3600 | 1 | 1030–2957 |
| 2 | 875–2595 | 2 | 800–3900 | 2 | 1183–3306 |
| 3 | 978–2858 | 3 | 900–4300 | 3 | 1343–3658 |
| 4 | 1068–3152 | 4 | 1000–4700 | 4 | 1511–4013 |
| 5 | 1147–3443 | 5 | 1100–5200 | 5 | 1687–4371 |
| 6 | 1219–3710 | 6 | 1300–5600 | 6 | 1868–4272 |
| 7 | 1282–3939 | 7 | 1400–6100 | 7 | 2053–5077 |
| 8 | 1339–4123 | 8 | 1600–6500 | 8 | 2239–5419 |
| 9 | 1389–4260 | 9 | 1800–7100 | 9 | 2423–5741 |
| 10 | 1433–4354 | 10 | 2100–7700 | 10 | 2603–6044 |
| 11 | 1470–4408 | 11 | 2400–8400 | 11 | 2775–6321 |
| 12 | 1502–4427 | 12 | 2700–8900 | 12 | 2935–6565 |
| 13 | 1527–4418 | 13 | 3100–9500 | 13 | 3080–6771 |
| 14 | 1555–4367 | 14 | 3300–10000 | 14 | 3205–6933 |
| 15 |  | 15 | 3500–10000 | 15 | 3306–7044 |
| 16 | 1574–4260 | 16 | 3400–9500 | 16 | 3379–7099 |
| 17~19 | 1577–4142 | 17 | 3200–8700 | 17 | 3423–7098 |
|  |  | 18 | 3100–7900 | 18 | 3441–7053 |
|  |  | 19 | 2900–7300 | 19 | 3439–6973 |
| ^1)^ IGFBP-3 reference values (Mean – 2SD to Mean + 2SD), determined by immunoradiometric assay (IDS), were calculated from 448 “normal” samples collected by Dr. Millet from ‘Centre Hospitalier Régional de la Citadelle’ in Liège, Belgium. This is detailed in the IDS Instruction for Use (CL-BC1014, Revision No. 07; effective date, 17 December 2015).  ^2)^ A reference range study was conducted using the IMMULITE IGFBP-3 kit, which included 85 neonatal samples (1-15 days old), and 1,499 pediatric and adult samples from an apparently healthy population. All RIs originally expressed in μg/mL were converted to ng/mL by multiplying 1,000. This information is provided in the Immulite 2000 IGFBP-3 insert (PIL2KIGF-15, dated 2018-03-15).  ^3)^ Expected values were obtained in a clinical study (CIM RD002970) involving over 1400 samples from subjects aged 17 years old or younger. Laboratories are advised to assess the transferability of the expected values to their own patient populations and establish their own reference ranges, if necessary. This is documented in the Elecsys IGFPB-3 insert (07574720190, dated May 2019 v1.0, Roche Diagnostics). | | | | | |

| Supplementary Table 4. IGFBP-3 (unit, ng/mL) female reference intervals according to manufacturers | | | | | |
| --- | --- | --- | --- | --- | --- |
| IRMA (IDS) ^1)^ | | Immulite 2000 (Siemens)^2)^ | | e801 (Roche)^3)^ | |
| Age (yr) | RIs | Age (yr) | RIs | Age (yr) | RIs |
| 0 | 689–2370 | - | - | 0 | 1086–3146 |
| 1 | 756–2412 | 1 | 700–3600 | 1 | 1228–3352 |
| 2 | 875–2595 | 2 | 800–3900 | 2 | 1420–3752 |
| 3 | 978–2858 | 3 | 900–4300 | 3 | 1614–4136 |
| 4 | 1068–3152 | 4 | 1000–4700 | 4 | 1810–4505 |
| 5 | 1147–3443 | 5 | 1100–5200 | 5 | 2007–4860 |
| 6 | 1219–3710 | 6 | 1300–5600 | 6 | 2203–5202 |
| 7 | 1282–3939 | 7 | 1400–6100 | 7 | 2397–5535 |
| 8 | 1339–4123 | 8 | 1600–6500 | 8 | 2588–5858 |
| 9 | 1389–4260 | 9 | 1800–7100 | 9 | 2774–6172 |
| 10 | 1433–4354 | 10 | 2100–7700 | 10 | 2951–6474 |
| 11 | 1470–4408 | 11 | 2400–8400 | 11 | 3116–6761 |
| 12 | 1502–4427 | 12 | 2700–8900 | 12 | 3264–7031 |
| 13 | 1527–4418 | 13 | 3100–9500 | 13 | 3392–7282 |
| 14 | 1555–4367 | 14 | 3300–10000 | 14 | 3501–7512 |
| 15 |  | 15 | 3500–10000 | 15 | 3589–7720 |
| 16 | 1574–4260 | 16 | 3400–9500 | 16 | 3658–7905 |
| 17~19 | 1577–4142 | 17 | 3200–8700 | 17 | 3705–8065 |
|  |  | 18 | 3100–7900 | 18 | 3734–8198 |
|  |  | 19 | 2900–7300 | 19 | 3745–8302 |
| ^1)^ IGFBP-3 reference values (Mean – 2SD to Mean + 2SD), determined by immunoradiometric assay (IDS), were calculated from 448 “normal” samples collected by Dr. Millet from ‘Centre Hospitalier Régional de la Citadelle’ in Liège, Belgium. This is detailed in the IDS Instruction for Use (CL-BC1014, Revision No. 07; effective date, 17 December 2015).  ^2)^ A reference range study was conducted using the IMMULITE IGFBP-3 kit, which included 85 neonatal samples (1-15 days old), and 1,499 pediatric and adult samples from an apparently healthy population. All RIs originally expressed in μg/mL were converted to ng/mL by multiplying 1,000. This information is provided in the Immulite 2000 IGFBP-3 insert (PIL2KIGF-15, dated 2018-03-15).  ^3)^ Expected values were obtained in a clinical study (CIM RD002970) involving over 1400 samples from subjects aged 17 years old or younger. Laboratories are advised to assess the transferability of the expected values to their own patient populations and establish their own reference ranges, if necessary. This is documented in the Elecsys IGFPB-3 insert (07574720190, dated May 2019 v1.0, Roche Diagnostics). | | | | | |
